# Association of Hospital Market Competition with Outcomes of Complex Cancer Surgery

**DOI:** 10.1245/s10434-024-15278-w

**Published:** 2024-04-18

**Authors:** Muhammad Musaab Munir, Selamawit Woldesenbet, Yutaka Endo, Mary Dillhoff, Susan Tsai, Timothy M. Pawlik

**Affiliations:** Department of Surgery, The Urban Meyer III and Shelley Meyer Chair for Cancer Research, The Ohio State University Wexner Medical Center and James Comprehensive Cancer Center, Columbus, OH USA

**Keywords:** Cannabis use, Mortality, Oncologic care, Substance use

## Abstract

**Background:**

The association of hospital market competition, financial costs, and quality of oncologic care has not been well-defined. This study sought to evaluate variations in patient outcomes and financial expenditures after complex cancer surgery across high- and low-competition markets.

**Methods:**

Medicare 100% Standard Analytic Files were used to identify patients with lung, esophageal, gastric, hepatopancreaticobiliary, or colorectal cancer who underwent surgical resection between 2018 and 2021. Data were merged with the annual hospital survey database, and the hospital market Herfindahl–Hirschman index was used to categorize hospitals into low- and high-concentration markets. Multi-level, multivariable regression models adjusting for patient characteristics (i.e., age, sex, comorbidities, and social vulnerability), year of procedure, and hospital factors (i.e., case volume, nurse-bed ratio, and teaching status) were used to assess the association between hospital market competition and outcomes.

**Results:**

Among 117,641 beneficiaries who underwent complex oncologic surgery, the mean age was 73.8 ± 6.1 years, and approximately one-half of the cohort was male (*n* = 56,243, 47.8%). Overall, 63.8% (*n* = 75,041) of the patients underwent care within a high-competition market. Notably, there was marked geographic variation relative to market competition. High versus low market-competition hospitals were more likely to be in high social vulnerability areas (35.1 vs 27.5%; *p* < 0.001), as well as care for racial/ethnic minority individuals (13.8 vs 7.7%; *p* < 0.001), and patients with more comorbidities (≥ 2 Elixhauser comorbidities: 63.1 vs 61.1%; *p* < 0.001). In the multivariable analysis, treatment at hospitals in high- versus low-competition markets was associated with lower odds of achieving a textbook outcome (odds ratio, 0.95; 95% confidence interval, 0.91–0.99; *p* = 0.009). Patients at high-competition hospitals had greater mean index hospitalization costs ($19,462.2 [16211.9] vs $18,844.7 [14994.7]) and 90-day post-discharge costs ($7807.8 [15431.3] vs $7332.8 [14038.2]) (both *p* < 0.001) than individuals at low-competition hospitals.

**Conclusions:**

Hospital market competition was associated with poor achievement of an optimal postoperative outcome and greater hospitalization costs.

**Supplementary Information:**

The online version contains supplementary material available at 10.1245/s10434-024-15278-w.

Centralization of surgical care has largely been driven by an improvement in observed clinical outcomes due to the volume-outcome relationship.^1,2^ This development is supported by many policy researchers who argue that merging health care systems helps to enhance the ability of clinical professionals to provide coordinated, interdisciplinary patient care. Furthermore, studies have demonstrated that regional centers with a higher surgical volume often experience lower complication rates and potentially reduced costs.^3,4^ In contrast, other research has suggested that increased competition among hospitals, indicating less market concentration, may lead to quicker adoption of innovative medical technologies.^5,6^ This shift toward greater regionalization in surgical care, partly driven by a national trend of hospital system consolidation, has raised questions about the complex relationship between hospital market competition and the quality and cost efficiency of health care services.^4^

The impact of hospital market competition on surgical outcomes is complex and not fully understood. For instance, competition has been linked to better management of medical conditions such as diabetes and reduced inpatient mortality for myocardial infarction.^7,8^ Nonetheless, the association between hospital competition and surgical outcomes is less clear.^9,10^ Some studies have indicated higher mortality in competitive environments for procedures such as kidney transplantation,^11^ whereas others suggest lower mortality for major surgical procedures in more competitive markets.^12^ These varied results may stem from limitations such as a limited sample of hospitals and procedures.

Additionally, the trend toward hospital consolidation and regionalization, partly driven by health care reforms, may be associated with both potential benefits and risks. Reforms aimed at improving quality of care and population health have led to changes, including hospital mergers and clinical integration of care across surgeons and hospitals.^13,14^ Interestingly, the Federal Trade Commission has stated that hospital consolidation may lead to higher prices, primarily due to increased bargaining power, reduced competition, decreased price transparency, patient steerage toward higher-priced facilities, and barriers to entry for new competitors.^15–17^ Nonetheless, other studies have found that consolidation also may result in cost savings and better outcomes through more efficient care delivery and integration.^18^ The evolving landscape complicates understanding of the relationship between hospital market competition, cost, and quality of care.

Many hospital services face competition, but complex, elective surgical procedures are notably more susceptible to this competitive environment. This may be due to variations in outcomes and patient demographics, as well as costs and quality of care across high- and low- competition markets. In addition, the incidence of complications associated with these surgical procedures tend to vary significantly, depending on the hospital in which the procedures are performed.^10^

Using recently accessible national data as well as validated outcome metrics and robust statistical techniques, the current study sought to evaluate whether hospital market competition was associated with surgical quality and cost of care among patients undergoing complex cancer surgery.

## Methods

### Data Source and Study Population

Data were derived from 100% Medicare Standard Analytic Files claims data obtained by the Centers for Medicare and Medicaid Services for fee-for-service beneficiaries enrolled in Medicare Parts A and B. Patients aged 65 or older who underwent a complex oncologic procedure were defined as individuals who underwent surgical resection for esophageal, lung, gastric, liver, pancreatic, biliary, colon, or rectal malignancies between 2018 and 2021. They were identified using diagnosis and procedure codes from the International Classification of Diseases, ninth and tenth revisions (ICD-9/10th). Index procedural codes were used to identify patients undergoing resection after establishment of the diagnosis.

The study excluded patients who were not enrolled in Medicare part A or B during the month of the surgical episode and individuals who received additional payments from a health maintenance organization (HMO). For patients who underwent more than one surgical procedure, the first operation was considered the analytic procedure. The study also excluded patients who underwent emergent or urgent index procedures. This study was approved, and informed consent for de-identified data was waived by the Institutional Review Board of the Ohio State University.

Hospital characteristics including region, teaching status, cancer program accreditation, and bed size were merged using the American Hospital Association annual survey data.^19^ Hospital procedure-specific volume was calculated and divided into tertiles (i.e., low, moderate, and high), as previously described.^20^ Data on a county-level social vulnerability index (SVI) were obtained from the Center for Disease Control and the Agency for Toxic Substances and Disease Registry. The SVI data are a recognized metric for evaluating the susceptibility of communities to external stressors.^21^ Patient comorbidities were defined using Elixhauser comorbidity software refined for ICD-10 Clinical Modification codes.^22^

Discharge location was categorized as home, home health agency, skilled nursing facility, and other (including other inpatient facilities, short-term care facilities, psychiatric facilities, and rehabilitation grouped together due to small sample sizes), as previously described.^20,23^

### Hospital Market Competition

Hospital market competition was defined using the Herfindahl-Hirschman index (HHI), as previously described.^10,12,24^ Each hospital in the United States is assigned a value on the HHI, ranging from 1000 to 10,000, with higher values indicating lower levels of competition. A hospital market was assigned according to health service areas (HSA), which are defined based on the hospital care patterns of fee-for-service Medicare beneficiaries and are referred to as “local healthcare markets.”^25,26^

The HHI was calculated based on the Kessler-McClellan approach, as a weighted average of geographic HHIs.^8,26^ Specifically, the HHI was calculated by multiplying the summed squared market shares of each hospital within a geographic market by 10,000. To calculate HSA-level HHI, a ZIP code-level HHI was first calculated by summing for each ZIP code every hospital–ZIP code combination’s squared share of total discharges in that ZIP code. A hospital-level HHI was calculated by summing for each hospital the ZIP code share of total discharges for that hospital multiplied by that ZIP code’s HHI. The HSA-level HHI then was calculated by taking a discharge-weighted average of all hospital HHIs in that HSA. This approach was consistent with prior literature that examined hospital concentration and Marketplaces.^8,27,28^

The hospitals were sorted into categories of high or low competition based on an index score cutoff of 4000, with higher values indicating low competition and lower values indicating high competition. This categorization was based on prior work from the National Bureau of Economic Research and designed to ensure a roughly equal distribution of patient groups in both high- and low-competition subgroups.^29^

To determine the market share of hospitals, discharge totals for the ZIP codes served by each hospital were obtained from the Hospital Service Area files of the Centers for Medicare and Medicaid Services.^30^ These files contain data on the number of Medicare discharges for each hospital from each ZIP code that it serves and include data up to 2017, whereas our clinical data start from 2018. During the study period from 2018 to 2021, the index values remained largely unchanged because the market dynamics in this sector were generally stable.^10^

### Outcomes of Interest

The primary outcome of interest was textbook outcome (TO), a composite quality-of-care metric, which was defined by the absence of all four of the following criteria: postoperative complication, extended stay, 90-day readmission, and 90-day mortality.^31,32^ Postoperative complications were determined using previously validated ICD-9/10th clinical modification and procedure codes.^31,32^ Hospital length of stay (LOS) was defined as time elapsed from the date of admission to the date of discharge, with an extended LOS identified as the index hospitalization with LOS greater than the 75th percentile. Readmission was defined as admission to any hospital within 90 days after discharge. Mortality was defined as death within 90-days after the index procedure.

The secondary outcomes included total Medicare expenditure in U.S. dollars during the index hospitalization period and 90 days after discharge. Expenditures were adjusted by wage index, indirect medical education, and disproportionate share hospital to allow for equitable comparisons.^33^

### Statistical Analysis

Continuous variables are reported as means with standard deviations and discrete variables as frequencies with percentages. Univariable comparisons were performed using the Wilcoxon rank-sum test for continuous variables and the chi-square test for categorical variables. Due to variability in baseline patient and facility characteristics among patients relative to hospital competition, entropy-balancing (EB) was performed to generate balanced groups for estimating the effects of market competition on outcomes. High- and low-competition hospitals were balanced for age, sex, race/ethnicity, social vulnerability, Elixhauser comorbidities, type of cancer, rural status, year of surgery, hospital region, hospital volume, nurse-bed ratio, hospital accreditation, hospital teaching status (chosen a priori).

Notably, the EB method calculates the weights for control units in a manner that ensures that the reweighted control group meets a predefined set of balance conditions, which are based on the sample’s distribution of covariates.^34^ Furthermore, an optimization problem aims to find a weight set that satisfies the balance constraints while being as similar as possible to the original weights to minimize information loss.^35^ The modification of unit weights efficiently addresses both systematic and random discrepancies in representation, providing a more effective correction for covariate imbalance.^34^ Importantly, entropy-balancing has demonstrated greater accuracy in estimation and demands less computational burden than alternative reweighting methods such as inverse probability-weighting and stabilized inverse probability-weighting.^36^

Multi-level, mixed-effect logistic regression analysis with robust standard errors and a random intercept for hospital was used to examine associations between hospital market competition and outcomes of interest. Multivariable generalized linear regression with gamma distribution and a log link was used to determine the association between hospital market competition and Medicare expenditures. Complete case analysis was used because less than 5% of patient hospitalizations had missing data.^37^

Results are reported as odds ratios (ORs) with 95% confidence intervals (CIs). All statistical analyses were derived from two-tailed tests, and a *p* value lower than 0.05 was considered statistically significant. The analyses were performed using STATA, version 18.0 (StataCorp, College Station, TX, USA).

## Results

### Baseline Characteristics

For 117,641 patients, a complex oncologic operation was performed to treat esophagus (*n* = 1660, 1.4%), lung (*n* = 42,144, 35.8%), gastric (*n* = 3377, 2.9%), hepatopancreaticobiliary (*n* = 11,028, 9.4%), and colorectal (*n* = 59,432, 50.5%) cancer. Overall, the mean patient age was 73.8 ± 6.1 years, and approximately half of the patients were female (*n* = 61,398, 52.2%). A majority of the patients had more than two comorbidities (*n* = 73,366, 62.4%) and resided in urban areas (*n* = 91,865, 78.3%). Most of the patients were white (*n* = 103,989, 88.4%), with smaller subsets of black (*n* = 6527, 5.5%), Hispanic (*n* = 892, 0.8%), and other race/ethnicity (*n* = 6233, 5.3%) patients (Table 1).

**Table 1:** Patient characteristics across high- and low-competition markets

Variables	Total (*n* = 117,641) *n* (%)	High competition (*n* = 75,041, 63.8%) *n* (%)	Low competition (*n* = 42,600, 36.2%) *n* (%)	*p* value^a^
Mean age (years)	73.8 ± 6.1	73.7 ± 6.1	73.9 ± 6.2	< 0.001^b^
Sex				0.037
Female	61,398 (52.2)	39,336 (52.4)	22,062 (51.8)	
Male	56,243 (47.8)	35,705 (47.6)	20,538 (48.2)	
Race/ethnicity				< 0.001^b^
White	103,989 (88.4)	64,689 (86.2)	39,300 (92.3)	
Black	6527 (5.5)	4944 (6.6)	1583 (3.7)	
Hispanic	892 (0.8)	681 (0.9)	211 (0.5)	
Other	6233 (5.3)	4727 (6.3)	1506 (3.5)	
Elixhauser comorbidities				< 0.001^b^
0–1	44,275 (37.6)	27,685 (36.9)	16,590 (38.9)	
≥2	73,366 (62.4)	47,356 (63.1)	26,010 (61.1)	
Cancer type				< 0.001^b^
Lung	42,144 (35.8)	28,005 (37.3)	14,139 (33.2)	
Esophageal	1660 (1.4)	1035 (1.4)	625 (1.5)	
Gastric	3377 (2.9)	2327 (3.1)	1050 (2.5)	
HPB	11,028 (9.4)	7908 (10.5)	3120 (7.3)	
CRC	59,432 (50.5)	35,766 (47.7)	23,666 (55.6)	
Social vulnerability index				< 0.001^b^
Low	39,785 (33.9)	24,516 (32.8)	15,269 (35.9)	
Moderate	39,560 (33.7)	24,019 (32.1)	15,541 (36.6)	
High	37,943 (32.4)	26,272 (35.1)	11,671 (27.5)	
Urban-rural continuum				< 0.001^b^
Urban	91,865 (78.3)	63,734 (85.2)	28,131 (66.2)	
Rural	25,420 (21.7)	11,073 (14.8)	14,347 (33.8)	
Year of surgery				0.071
2018	31,820 (27.0)	20,187 (26.9)	11,633 (27.3)	
2019	33,719 (28.7)	21,691 (28.9)	12,028 (28.2)	
2020	26,780 (22.8)	17,000 (22.7)	9780 (23.0)	
2021	25,322 (21.5)	16,163 (21.5)	9159 (21.5)	
Patient region				< 0.001
Midwest	27,121 (23.1)	15,848 (21.1)	11,273 (26.5)	
Northeast	23,453 (19.9)	15,918 (21.2)	7535 (17.7)	
South	47,103 (40.0)	30,005 (40.0)	17,098 (40.1)	
West	19,963 (17.0)	13,269 (17.7)	6694 (15.7)	
Discharge location				< 0.001*
Home health agency	7384 (6.3)	5222 (7.0)	2162 (5.1)	
Home	21,524 (18.3)	14,352 (19.1)	7172 (16.8)	
Other	85,692 (72.8)	53,464 (71.2)	32,228 (75.7)	
Skilled nursing facility	3041 (2.6)	2003 (2.7)	1038 (2.4)	

HPB, Hepatopancreatobiliary; CRC, Colorectal cancer

^a^Statistical tests performed: chi-square test of independence; Wilcoxon rank-sum test

^b^Statistically significant: *p* < 0.05

**Fig. 1:**
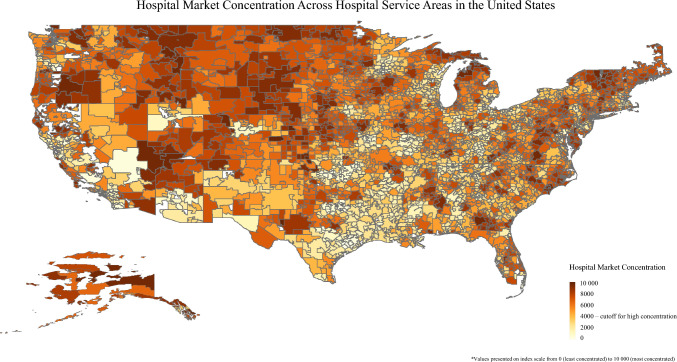
Geographic distribution of hospital market concentration measured by the Herfindahl-Hirschman index across hospital services areas in the United States

Among the Medicare beneficiaries who underwent an operation of interest, 63.8% (*n* = 75,041) received care in a high-competition market. Notably, there was marked geographic variation relative to market competition (Fig. 1). Relative to low-competition hospitals, high-competition facilities were more likely to be in high social vulnerability areas (35.1 vs. 27.5%; *p* < 0.001) and to care for racial/ethnic minority individuals (13.8 vs 7.7%; *p* < 0.001) as well as patients with more comorbidities (≥ 2 Elixhauser comorbidities: 63.1 vs 61.1%; *p* < 0.001).

Interestingly, high-competition hospitals performed a higher rate of lung (37.3 vs 33.2%), gastric (3.1 vs 2.5%), and hepatopancreaticobiliary (10.5 vs 7.3%) procedures than low-competition hospitals. In contrast, low-competition centers performed higher rates of esophageal (1.4 vs 1.5%) and colorectal (47.7 vs 55.5%) procedures than high-competition hospitals (*p* < 0.001). Hospitals in high-competition areas differed in their discharge locations from those in low-competition areas. Notably, hospitals facing high competition tended to discharge patients either to their homes (26.1 vs 21.9%) or to skilled nursing facilities (2.7 vs 2.4%) more frequently than hospitals in less competitive environments (both *p* < 0.001).

Among the 3889 hospitals included in the analytic cohort, 2318 (59.6%) were in high-competition markets, and 1571 (40.4%) were in low-competition markets. High-competition hospitals were more likely than low-competition hospitals to be teaching institutions (9.1 vs 5.3%) and to have cancer programs accredited by the Joint Commission (74.4 vs 58.5%) (both *p* < 0.001). High-competition hospitals also had a greater mean number of beds than low-competition hospitals (203 ± 219 vs 137 ± 180; *p* < 0.001). Most high-competition hospitals were in the southern part of the United States (*n* = 946, 41.5%), whereas most low-competition hospitals were in the Midwest (*n* = 558, 35.5%). High-competition hospitals had a higher mean nurse-to-bed ratio (1.4 ± 0.8) than low-competition hospitals (1.3 ± 0.8) (*p* = 0.022; Table 2).

**Table 2:** Hospital-level characteristics across high- and low-competition markets

Variables	Total (*n* = 3889) *n* (%)	High competition (*n* = 2318, 59.6%) *n* (%)	Low competition (*n* = 1571, 40.4%) *n* (%)		*p* value^a^
Hospital region				< 0.001^b^	
Midwest	1181 (30.7)	623 (27.3)	558 (35.5)		
Northeast	529 (13.7)	286 (12.5)	243 (15.5)		
South	1404 (36.5)	946 (41.5)	458 (29.2)		
West	738 (19.2)	426 (18.7)	312 (19.9)		
Hospital volume				0.231	
Low	2387 (61.4)	1447 (62.4)	940 (59.8)		
Moderate	1030 (26.4)	602 (26.0)	428 (27.2)		
High	472 (12.1)	269 (11.6)	203 (12.9)		
Mean no. of beds	176 ± 207	203 ± 219	137 ± 180		
Teaching hospital	293 (7.5)	210 (9.1)	83 (5.3)	< 0.001^b^	
Cancer program accreditation by the Joint Commission	2574 (67.9)	1671 (74.4)	903 (58.5)	< 0.001^b^	
Mean nurse-bed ratio	1.4 ± 0.8	1.4 ± 0.8	1.3 ± 0.8	0.022^b^	

^a^Statistical tests performed: χ2 test of independence; Wilcoxon rank-sum test

^b^Statistically significant: *p* < 0.05

### Hospital Market Competition and Outcomes

Overall, a TO was achieved for 60.8% (*n* = 71,544) of the patients. The patients who underwent care at a high-competition hospital (*n* = 45,466, 60.6%) were slightly less likely to experience this composite outcome than the patients who underwent care at a low-competition hospital (*n* = 26,078, 61.2%; *p* = 0.034). In turn, the patients who received care at high-competition hospitals and those who received care at low-competition hospitals differed in terms of perioperative complications (18.2 vs 17.3%) and readmission at 90 days (20.4 vs 19.8%) (both *p* < 0.05). Notably, the patients at high-competition hospitals had greater mean index hospitalization costs ($19,462.2 ± $16,211.9 vs $18,844.7 ± $14,994.7]) and 90-day post-discharge costs ($7807.8 ± $15,431.3] vs $7332.8 ± $14,038.2]) (both *p* < 0.001) than the individuals at low-competition hospitals (Table 3).

**Table 3:** Association between hospital market competition and overall outcomes

Outcomes	Unadjusted analysis		Entropy-balanced analysis		
	High competition	Low competition	OR	95% CI	*p* value
90-Day mortality	3374 (4.5)	2097 (4.9)	0.97	0.89–1.05	0.459
90-Day readmission	15,228 (20.4)	8441 (19.8)	1.04	1.00–1.08	0.059
Perioperative complications	13,661 (18.2)	7373 (17.3)	1.11	1.06–1.17	<0.001^a^
Extended stay	11,800 (15.7)	6585 (15.5)	1.04	0.98–1.10	0.213
Textbook outcome	45,466 (60.6)	26,078 (61.2)	0.95	0.91–0.99	0.009^a^
Mean index expenditure	19,462.2 ± 16,211.9	18,844.7 ± 14,994.7	1.07	0.72–1.58	0.738
Mean 90-day expenditure	7807.8 ± 15,431.3	7332.8 ± 14,038.2	1.56	1.17–2.09	0.003^b^

*OR*, Odds ratio; *CI*, Confidence interval

Multi-level, Mixed effect model balanced for age, sex, race/ethnicity, social vulnerability, Elixhauser comorbidities, type of cancer, rural status, year of surgery, hospital region, hospital volume, nurse-bed ratio, hospital accreditation, hospital teaching status

^a^Statistically significant: *p* < 0.05

In the multivariable analysis, after balancing for other risk factors, treatment at hospitals in high-competition markets was associated with greater odds of perioperative complications (OR, 1.11; 95% CI, 1.06–1.17; *p* < 0.001) than treatment in low-competition markets. In contrast, the odds of achieving a TO were lower (OR, 0.95; 95% CI, 0.91–0.99; *p* = 0.009), and the mean Medicare expenditures for the index surgical admission did not differ (OR, 1.07; 95% CI, 0.72–1.58; *p* = 0.738). Interestingly, the patients who underwent care at high-competition hospitals had higher risk-adjusted 90-day expenditures (OR, 1.56; 95% CI, 1.17–2.09; *p* = 0.003). The multivariable analysis showed no difference in the odds of an extended stay, mortality, or readmission within 90 days (both *p* > 0.05).

**Fig. 2:**
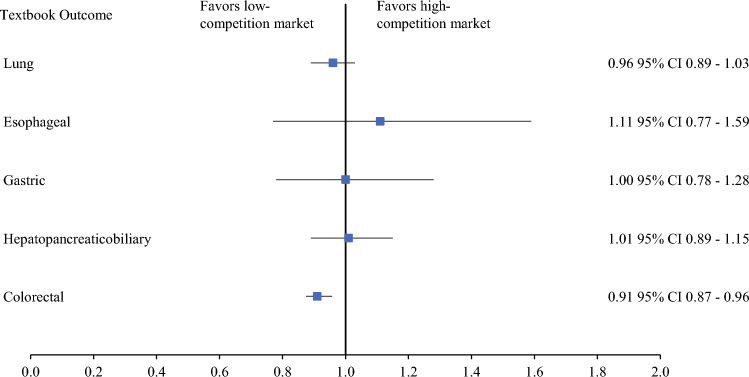
Adjusted association between hospital competition and textbook outcomes among patients undergoing a range of high-risk surgical procedures for cancer

Due to heterogeneity between oncologic procedure types, stratified analysis was performed for each cancer. The association between treatment at high-competition hospitals and TO persisted only for colorectal operations (colorectal: OR, 0.91; 95% CI, 0.87–0.96; *p* < 0.001; Fig. 2). There was marked variation in the association between hospital competition and individual outcome parameters across cancer types (Table S1). Notably, high-competition hospitals were associated with greater odds of perioperative complications (OR, 1.14; 95% CI, 1.07–1.21), an extended stay (OR, 1.08; 95% CI, 1.00–1.17), and readmission with 90 days (OR, 1.07; 95% CI, 1.01–1.14) for colorectal procedures (all *p* < 0.05).

## Discussion

Higher surgical volume in hospitals has been linked to better patient outcomes, such as reduced mortality, fewer complications, and enhanced survival among patients with cancer.^1,38^ Critics of health care system consolidation contend, however, that reduced competition fosters a seller’s market, allowing hospitals to dictate prices and consequently limit patient options for receiving care.^39^ Moreover, recent studies examining the relationship between hospital competition and perioperative outcomes have yielded conflicting results.^9–12^

To date, few studies have examined whether a rise in interhospital market competition (thereby reducing market concentration) is associated with adverse clinical and financial outcomes for patients and payers. Therefore, the current work was important because we specifically sought to evaluate whether hospital market competition was associated with surgical quality and cost of care among patients undergoing complex cancer surgery.

Using a nationally representative cohort, this study found that hospital market competition was associated with higher Medicare expenditures and a lower likelihood of an optimal postoperative outcome (i.e., TO) after a range of complex oncologic procedures. Furthermore, high-competition hospitals more often served racial/ethnic minority individuals, individuals residing in areas with high social vulnerability, and patients with multiple comorbidities. Taken together, these findings call into question the widespread notion that hospital competition improves quality of care, especially among patients with cancer who undergo surgical resection.

Despite notable improvements in cancer care delivery at the national level, considerable inequities persist among various groups within society. Prior research has highlighted concerns about the disparity in patient health status among hospitals in high-competition versus low-competition areas.^10,40^ For instance, a study by Tang et al.^41^ on unruptured intracranial aneurysms demonstrated that hospitals in more competitive areas tended to care for older patients with a higher risk of preoperative mortality.

Another study focusing on kidney transplantation illustrated that hospitals in competitive markets were more likely to operate on individuals with numerous comorbidities and a higher likelihood of adverse clinical outcomes.^11^ Similarly, previous work by our own group noted that hospitals facing more competition served patients with multiple comorbidities and a high mortality risk prediction.^12^ Interestingly, a recent study by Thumma et al.^10^ noted that older patients and individuals from racial/ethnic minority backgrounds more often underwent care at high-competition hospitals.

The current study built on previous work by examining the role of social determinants of health (SDoH) relative to market competition. Specifically, we used the social vulnerability index, a validated composite measure of SDoH developed by the Centers for Disease Control and Prevention (CDC) using 16 census variables including poverty, lack of vehicular access, and crowded housing to measure community resilience and disaster preparedness.^21^ Notably, in addition to racial/ethnic minority individuals and patients with multiple comorbidities, hospitals in highly competitive environments tended to care for patients residing in areas with high social vulnerability. In turn, the data strongly suggest that racial/ethnic minority individuals face a “double disparity hit” because their racial/ethnic background coupled with a greater likelihood of residing in socially vulnerable areas make them even more susceptible to receiving complex cancer care at high-competition hospitals.

Recent trends in regionalization efforts have focused largely on patients undergoing complex cancer surgery, resulting in marked changes including hospital mergers, variations in payment schemes, and clinical integration of care across surgeons and hospitals.^13,14,38,42^ This increase in hospital consolidation may lead to higher costs of care due to greater market power without an accompanying improvement in surgical quality.^4,16,17,43^ Conversely, it also may result in cost reductions and improved clinical outcomes due to more streamlined care delivery.^18^ As a result, previous research examining the delivery of high-quality surgical care relative to hospital competition has yielded equivocal results. One study that used the Nationwide Inpatient Sample dataset previously reported an association between high hospital competition and lower mortality across major surgical procedures, including a lower incidence of failure-to-rescue after complications such as coronary artery bypass grafts.^12^

In contrast, a study on patients undergoing renal transplantation demonstrated that highly competitive hospital markets reported higher mortality, graft failure, and a greater risk of adverse outcomes.^11^ The current study adds to the literature by using a nationally representative cohort to examine the association between hospital market competition and patients who underwent high-risk surgical resection for a malignant diagnosis. Notably, the current study demonstrated a significant difference in confounder-adjusted risk of a validated, composite quality measure (i.e., TO) among patients undergoing complex oncologic procedures. In fact, patients who underwent surgical care for cancer at a high-competition hospital had higher rates of perioperative complications, resulting in poor achievement of a postoperative TO.

Although the exact mechanism for these findings is undeniably complex and multifaceted, hospitals facing greater competition may serve as the destination site for patients with multiple comorbidities and high social vulnerability.^10,40^ In addition, high-competition hospitals may be more inclined to undertake surgical procedures that might not be available in low-competition markets, often opting to operate on patients with a higher risk of worse outcomes, as observed in both this study and prior work.^10,40,44^

The current study observed a modest difference in index hospitalization costs between patients undergoing care across different hospital markets in unadjusted analysis, which disappeared after adjustment for confounders. This may have been due in part to the slight rise in perioperative complications among the patients undergoing care at high-competition hospitals. In addition, specialized centers with greater market consolidation (i.e., those reflective of high regionalization with less interhospital competition) providing high-risk surgical care have adopted enhanced recovery after surgery pathways, which has been associated with reduced hospital costs.^24,45,46^ Interestingly, after confounder adjustment, high-competition hospitals remained associated with 90-day post-discharge Medicare expenditures. This finding could be partly attributable to the marginal increase in 90-day readmission observed among socially vulnerable and racial/ethnic minority individuals treated at hospitals in highly competitive markets.

The current work holds implications for health care providers, organizations, and policymakers interested in the intersection between hospital market competition and delivery of cost-efficient, high-quality surgical care. For patients with cancer, an understanding of these dynamics may help inform decisions in choosing a surgeon. In particular, hospital competition has a varying effect on the “ideal” location to receive complex, oncologic care.^47^ Policymakers may consider these results in their efforts to optimize financial and health policies related to hospital competition. Given the potential link between hospital competition and adverse clinical outcomes, high-competition hospitals may represent a target for quality improvement of surgical care, particularly in the context of cancer.

Several limitations need to be considered when the results of this study are interpreted. The current study used a large administrative dataset that may have inherent constraints because it relied on diagnosis and procedure codes from billing data to infer clinical information. Although the Medicare claims data have been validated for large-scale quality assessment among patients with cancer, the dataset precludes the ability to differentiate between curative-intent resection and staging laparoscopic procedures with potential therapeutic effect.^48^ Moreover, the findings of the current study were limited to the specific procedures examined as well as to Medicare beneficiaries older than 65 years and may not be generalizable to other complex surgical procedures and patient populations. Similarly, the Medicare dataset may not represent the ideal population for evaluating quality of care and costs. For instance, these data cannot consider major differences in characteristics among hospitals or hospital systems stemming from market competition status, such as negotiated reimbursement rates and cost structures. Health care diverges from conventional business norms by prioritizing volume-based incentives and typically undervalues costs and care quality within fee-for-service models, with market competition driven primarily by reputation, marketing tactics, and negotiations with payers. Nonetheless, the usage of Medicare data provided a unique resource of patient- and hospital-level identifiable information, offering a more geographically representative sample than other studies. Moreover, most of the complex oncologic procedures included were more prevalent among the older population in the United States. Furthermore, admissions for selected operations were assessed rather than diagnosis of incident malignancy.

In addition, the current study relied on a single measure of hospital market competition, which may have not fully encompassed how market dynamics affect surgical outcomes. Despite the use of robust statistical methods to adjust for observed confounders, the possibility of bias from unmeasured confounders has been an inherent limitation of retrospective observational studies.^49^

In conclusion, the current study demonstrated that hospital market competition was associated with surgical quality and cost of care after complex cancer surgery. High-competition hospitals were more likely to care for sicker and more vulnerable patient populations than low-competition hospitals. These data highlight the need to focus on improving competition-related differences to improve quality of surgical care among patients with cancer.

### Supplementary Information

Below is the link to the electronic supplementary material.

Supplementary file1 (DOCX 41 kb)

## Data Availability

The data for this study were obtained from the Medicare Standard Analytic Files. There are restrictions to the availability of these data, which are used under license for this study. Data can be accessed with permission from the Centers for Medicare and Medicaid Services.
