# Supplementary material for: Association of Hospital Market Competition with Outcomes of Complex Cancer Surgery
Source: Ann Surg Oncol. 2024 Apr 18;31(7):4371–80. doi: 10.1245/s10434-024-15278-w (PMC11164796; doi:10.1245/s10434-024-15278-w)
Supplement: Supplementary file 1 — Supplementary file1 (DOCX 41 kb) [file 10434_2024_15278_MOESM1_ESM.docx]

**Supplemental Table 1.** Association between Hospital Market Competition and Postoperative Outcomes relative to oncologic procedure type.

| **Outcomes** | **Unadjusted Analysis** | | **Entropy-Balanced Analysis** | | |
| --- | --- | --- | --- | --- | --- |
|  | **High Competition** | **Low Competition** | **OR** | **95% CI** | ***p*-value** |
| **Lung (n=42,144)** |  |  |  |  |  |
| 90-day mortality | 883 (3.2%) | 505 (3.6%) | 0.99 | 0.85-1.15 | 0.887 |
| 90-day readmission | 4,602 (16.4%) | 2,308 (16.3%) | 1.07 | 0.98-1.16 | 0.117 |
| Perioperative complications | 4,215 (15.1%) | 2,115 (15.0%) | 1.13 | 1.03-1.24 | 0.012 |
| Extended length-of-stay | 5,235 (18.7%) | 2,966 (21.0%) | 1.00 | 0.91-1.10 | 0.946 |
| Textbook outcome | 17,714 (63.3%) | 8,720 (61.7%) | 0.96 | 0.89-1.03 | 0.232 |
| Index expenditure | 18723.2 ± 14575.0 | 18796.4 ± 14268.2 | 0.92 | 0.48-1.73 | 0.787 |
| 90-day expenditure | 6775.4 ± 14483.8 | 6655.2 ± 13203.7 | 1.69 | 1.07-2.66 | 0.003 |
| **Esophageal (n=1660)** |  |  |  |  |  |
| 90-day mortality | 115 (11.1%) | 73 (11.7%) | 1.05 | 0.59-0.85 | 0.862 |
| 90-day readmission | 297 (28.7%) | 190 (30.4%) | 1.01 | 0.71-1.45 | 0.950 |
| Perioperative complications | 417 (40.3%) | 220 (35.2%) | 1.09 | 0.76-1.56 | 0.647 |
| Extended length-of-stay | 237 (22.9%) | 125 (20.0%) | 0.99 | 0.63-1.57 | 0.980 |
| Textbook outcome | 422 (40.8%) | 262 (41.9%) | 1.11 | 0.77-1.59 | 0.575 |
| Index expenditure | 40982.4 ± 41922.9 | 39032.1 ± 44672.0 | 0.02 | 0.00-28.66 | 0.293 |
| 90-day expenditure | 15257.8 ± 29392.7 | 13580.4 ± 26815.8 | 3.49 | 0.08-145.75 | 0.511 |
| **Gastric (n=3377)** |  |  |  |  |  |
| 90-day mortality | 160 (6.9%) | 85 (8.1%) | 1.43 | 0.93-2.20 | 0.100 |
| 90-day readmission | 536 (23.0%) | 258 (24.6%) | 1.02 | 0.77-1.34 | 0.900 |
| Perioperative complications | 552 (23.7%) | 286 (27.2%) | 0.99 | 0.76-1.31 | 0.968 |
| Extended length-of-stay | 409 (17.6%) | 194 (18.5%) | 1.44 | 1.01-2.02 | 0.039 |
| Textbook outcome | 1,310 (56.3%) | 554 (52.8%) | 1.00 | 0.78-1.28 | 0.972 |
| Index expenditure | 24885.1 ± 22990.2 | 25896.3 ± 24503.2 | 1.05 | 0.10-10.58 | 0.967 |
| 90-day expenditure | 9879.3 ± 18858.2 | 9870.6 ± 17804.0 | 1.67 | 0.30-9.40 | 0.559 |
| **HPB (n=11,028)** |  |  |  |  |  |
| 90-day mortality | 518 (6.6%) | 242 (7.8%) | 1.08 | 0.85-1.35 | 0.539 |
| 90-day readmission | 2,355 (29.8%) | 951 (30.5%) | 1.03 | 0.90-1.19 | 0.655 |
| Perioperative complications | 2,042 (25.8%) | 774 (24.8%) | 1.11 | 0.95-1.30 | 0.181 |
| Extended length-of-stay | 1,501 (19.0%) | 561 (18.0%) | 1.31 | 1.08-1.59 | 0.006 |
| Textbook outcome | 3,869 (48.9%) | 1,515 (48.6%) | 1.01 | 0.89-1.15 | 0.840 |
| Index expenditure | 27754.0 ± 22596.4 | 26577.6 ± 22719.8 | 3.95 | 1.04-15.0 | 0.043 |
| 90-day expenditure | 11116.4 ± 19429.1 | 10978.8 ± 18338.4 | 0.98 | 0.32-3.00 | 0.972 |
| **Colorectal (n=59,432)** |  |  |  |  |  |
| 90-day mortality | 1,698 (4.7%) | 1,192 (5.0%) | 0.99 | 0.89-1.10 | 0.880 |
| 90-day readmission | 7,498 (21.0%) | 4,734 (20.0%) | 1.07 | 1.01-1.14 | 0.013 |
| Perioperative complications | 6,435 (18.0%) | 3,978 (16.8%) | 1.14 | 1.07-1.21 | <0.001 |
| Extended length-of-stay | 4,418 (12.4%) | 2,739 (11.6%) | 1.08 | 1.00-1.17 | 0.043 |
| Textbook outcome | 22,151 (61.9%) | 15,027 (63.5%) | 0.91 | 0.87-0.96 | <0.001 |
| Index expenditure | 17243.0 ± 12323.6 | 17013.0 ± 10583.6 | 1.14 | 0.81-1.62 | 0.446 |
| 90-day expenditure | 7481.9 ± 13869.3 | 6953.6 ± 12949.7 | 1.72 | 1.22-2.42 | 0.002 |

**^*^** ^Statistically significant:^ *^p^*^­-value < 0.05
Abbreviations: HPB, hepatopancreaticobiliary
Multi-level, mixed effect model balanced for age, sex, race/ethnicity, social vulnerability, Elixhauser comorbidities, type of cancer, rural status, year of surgery, hospital region, hospital volume, nurse-bed ratio, hospital accreditation, hospital teaching status.^
